# Narrative Ability in Autism and First-Degree Relatives

**DOI:** 10.1007/s10803-024-06424-0

**Published:** 2024-07-26

**Authors:** Kritika Nayar, Emily Landau, Gary E. Martin, Cassandra J. Stevens, Jiayin Xing, Pirog Sophia, Janna Guilfoyle, Peter C. Gordon, Molly Losh

**Affiliations:** 1 Northwestern University, 2240 Campus Drive, Frances Searle Building, #2-366, Evanston, IL 6020 USA; 2 St. John’s University, Queens, NY USA; 3 University of North Carolina at Chapel Hill, Chapel Hill, NC USA

**Keywords:** Narrative, Pragmatic language, Broad autism phenotype, Autism, Siblings, Eye tracking

## Abstract

Narrative is an important communication skill for sharing personal experiences and connecting with others. Narrative skills are often impacted in autism spectrum disorder (ASD) and have important consequences for social interactions and relationships. Subtle differences in narrative have also been reported among first-degree relatives of autistic individuals, suggesting that narrative may also be an etiologically important language-related skill that is influenced by genes associated with ASD. This study examined narrative ability and related visual attention during narration in ASD and first-degree relatives of individuals with ASD (siblings and parents) to understand how narrative and related attentional styles may be variably impacted across the spectrum of ASD genetic influence. Participants included 56 autistic individuals, 42 siblings of autistic individuals, 49 controls, 161 parents of autistic individuals, and 61 parent controls. Narratives were elicited using a wordless picture book presented on an eye tracker to record concurrent gaze. Findings revealed parallel patterns of narrative differences among ASD and sibling groups in the use of causal language to connect story elements and the use of cognitive and affective language. More subtle differences within the domain of causal language were evident in ASD parents. Parallel patterns in the ASD and sibling groups were also found for gaze during narration. Findings implicate causal language as a critical narrative skill that is impacted in ASD and may be reflective of ASD genetic influence in relatives. Gaze patterns during narration suggest similar attentional mechanisms associated with narrative among ASD families.

## Introduction

Narrative, or storytelling, is a critical tool used to share experiences and connect with others in our social world. Narrative skill is often impacted in autism spectrum disorder (ASD) and can influence participation in everyday activities such as initiating and maintaining reciprocal conversations and building relationships (Capps et al., 2000; Colle et al., 2008; Losh & Capps, 2003, 2006; Reilly et al., 1998; Tager-Flusberg, 1995). Key differences in narrative skill reported in ASD include reduced mention of internal states (Diehl et al., 2006; King et al., 2014; Losh & Capps, 2003; Siller et al., 2014), omission of key story elements (e.g., setting, central conflict, resolution) (Barnes & Baron-Cohen, 2012; Lombardo et al., 2007; Losh & Capps, 2003), inclusion of tangential story details that detract from overarching plot and themes of the story (Lam & Yeung, 2012; Tager-Flusberg & Anderson, 1991), and limited use of causal language to connect and interpret the meaning of narrated experiences (Diehl et al., 2006; Losh & Capps, 2003, 2006; Tager-Flusberg & Sullivan, 1995). Differences in the use of these narrative devices are significant, as each play important roles in supporting contingent, thematically coherent, and meaningful social communication (e.g., Bruner, 1987; Ochs & Capps, 2009). Narrative skills also play an important role in the formation and recounting of memories and construction of mental schema (Baixauli et al., 2016). As such, differences in narrative in ASD can limit access and interaction with the social world (Losh & Capps, 2003, 2006), with important implications for impacts on daily life.

Prior work in which participants freely narrated a wordless picture book (Diehl et al., 2006; Losh & Capps, 2003, 2006; Tager-Flusberg & Sullivan, 1995) found that although autistic individuals were as likely as their typically developing peers to establish and maintain the story’s central search theme, they included fewer story components (e.g., description of setting, resolution) and causal attributions of the characters’ internal states and behaviors (e.g., “the boy was sad *because he realized* that his frog was missing”). Utilizing computational methods to examine narrative competence in different contexts, Lee et al. (2020) and Losh and Gordon (2014) also found that relative to controls, individuals with ASD produced poorer quality narratives during a less structured task, but similar quality narratives during a more structured task. These findings suggest that in the absence of structure, autistic individuals have greater difficulty producing coherent narratives. Studying narratives allows for the discovery of specific language patterns and can enrich understanding of how individuals with ASD encode salient information through attentional processes and use that information to inform storytelling.

Subtle differences in narrative ability (along with broader pragmatic, or social language differences) have also been documented among parents and siblings of autistic individuals (Ben-Yizhak et al., 2011; Di Michele et al., 2007; Landa et al., 1991, 1992; Lee et al., 2020; Losh et al., 2008a; Miller et al., 2015; Patel et al., 2020, 2022, 2023; Piven et al., 1997a, b), suggesting a genetic influence on narrative abilities associated with ASD. Strong evidence suggests that differences in pragmatic language constitute a principal component of the broad autism phenotype (BAP), a core set of subclinical personality and language features in clinically unaffected relatives thought to reflect ASD-related genetic variation (Bernier et al., 2012; Bolton et al., 1998; Klusek et al., 2014a, 2021; Losh et al., 2008b, 2012; Losh & Piven, 2007; Nayar et al., 2021; Piven et al., 1997a, b). Regarding narrative skills in particular, reduced narrative complexity and coherence has been reported among subgroups of parents of autistic individuals (Landa et al., 1991; Lee et al., 2020).

Although narrative competence has not extensively been examined in siblings of autistic individuals, studies of parent-reported and standardized tests of pragmatic language abilities more broadly have reported variable findings (cf. Ben-Yizhak et al., 2011; Bishop et al., 2006; Gangi et al., 2021; Greenslade et al., 2019; Miller et al., 2015; Pilowsky et al., 2003). Recent evidence from a meta-analytic study identified a small effect size indicating poorer pragmatic language abilities in siblings of autistic individuals compared to controls (Roemer, 2021). Two studies using examiner ratings of pragmatic language in school-age children revealed a step-wise pattern, with autistic individuals demonstrating the most difficulty, and clinically unaffected ASD siblings showing pragmatic skills intermediate to the ASD and control groups (Gangi et al., 2021; Greenslade et al., 2019). Importantly, a significant portion of the literature on pragmatic abilities of siblings has been conducted on infants and toddlers, focusing on early developmental periods before the emergence of complex pragmatic skills and often relying on parent-report measures (Iverson et al., 2018; LeBarton & Iverson, 2016; Miller et al., 2015; Toth et al., 2007; West et al., 2020), motivating the present study’s focus on school-age, adolescent, and adult individuals where pragmatics abilities are well-developed in typical development (Bamberg & Reilly, 1996; Berman & Slobin, 1994; Ervin-Tripp et al., 1990). As such, the present study will be one of the first to extend work to children and adult siblings of individuals with ASD.

Pragmatic language in the BAP could be an etiologically significant phenotype providing clues to the complex and multifactorial causes of ASD (Losh et al., 2008b; Nayar et al., 2021) and may offer a promising avenue to disentangle gene-brain-behavior relationships. Evidence of visual attention differences associated with narrative skill in ASD and their parents further underscores the potential biological significance of narrative studies in ASD. Specifically, given the automatic and rapid nature of eye movements, analysis of visual attention represents an intermediate link between brain and behavior that can reveal cognitive differences reflecting neurobiology (Adolphs, 2010; Dalton et al., 2005; Kanwisher et al., 1997; Sabatinelli et al., 2011; Yucel et al., 2015). For example, visual attention influences how an individual interprets the environment, is associated with an array of neuropsychological abilities including cognition, sensory regulation, and language processing, and is strongly rooted in core neurological functions (Altmann & Kamide, 2007; Eckstein et al., 2017; Lewis & Brooks-Gunn, 1981; Stechler & Latz, 1966).

Differences in social attention and visual attentional patterns have been repeatedly documented in ASD (Chita-Tegmark, 2016; Frazier et al., 2017; Król & Król, 2020; Landry & Bryson, 2004; Manyakov et al., 2018; Micai et al., 2017; Nayar et al., 2018, 2022; Papagiannopoulou et al., 2014; Sacrey et al., 2013; Sasson et al., 2008), with similar, but more subtle differences noted in subgroups of parents and siblings of individuals with ASD (Adolphs et al., 2008; Bhat et al., 2010; Canu et al., 2021; Chawarska et al., 2016; Dalton et al., 2007; Elsabbagh et al., 2013; Groen et al., 2012; Hogan-Brown et al., 2014; Kleberg et al., 2019; Merin et al., 2007; Nayar et al., 2018, 2022; Wagner et al., 2018). Studies utilizing concurrent eye tracking and narrative elicitation have reported evidence that such visual attention differences may importantly contribute to differences in narrative (Lee et al., 2020; Nayar et al., 2018). Specifically, Lee et al. (2020) found that autistic individuals and their parents attended more to the setting of a complex image than controls, and that visual attention to the setting or bodies of characters was associated with poorer narrative quality. Although not specifically examining narration, evidence also exists showing that joint attention during infancy predicts school-age pragmatic communication in ASD siblings (Greenslade et al., 2019). Taken together, evidence supports the hypothesis that differences in attention might in part drive some of the social communication patterns observed in ASD, and perhaps among clinically unaffected relatives as well.

### Current Study

The present study aimed to characterize narrative skills in ASD and first-degree relatives to determine whether similar profiles may be evident among clinically unaffected relatives. A detailed hand-coding scheme (utilizing a granular, scene-by-scene coding system that taps into elements including narrative quality, emotional inference, and detail orientation) was applied to narratives from autistic individuals, their siblings, and parents and respective control groups during free narration of a wordless picture book. In addition, this study investigated social attention differences and potential relationships to narrative skills using traditional eye-tracking measures (fixations and fixation duration towards social or non-social stimuli), as well as analysis of dynamic patterns of visual attention that are often reflected in the real world (e.g., Nayar et al., 2022), and may help to illuminate visual attention patterns related to social-communicative features of ASD and the BAP.

Differences in narrative were predicted to be greatest in the ASD group, and evidenced through narrative elements reflecting overall structure, as well as more fine-grained narrative features serving to connect story elements, such as causal explanations and explanations of protagonists’ thoughts and emotions. More subtle differences in these narrative elements were predicted in the sibling and parent groups. Eye-tracking analyses examining links between visual attention and narrative elements were exploratory.

## Methods

### Participants

This study included a total of 369 participants, including 56 autistic individuals (ASD Group), 42 siblings of autistic individuals (ASD Sibling Group), 161 parents of autistic individuals (ASD Parent Group), and respective controls (49 Controls for the ASD and ASD Sibling Groups, and 61 Parent Controls). This resulted in 58 parent-child dyads in the ASD or ASD Sibling Groups, 20 parent-child dyads in the Control Group, and 2 ASD sibling-ASD proband dyads. A portion of this group (33 ASD, 148 ASD parents, 38 controls, and 58 parent controls) were included in a prior publication (Lee et al., 2020). Fifty newly tested participants (23 autistic individuals, 11 controls, 13 parents of autistic individuals, 3 parent controls) were added for analyses of novel hand coding, additional gaze characterization, and comparison in relation to the newly tested ASD Sibling Group. Participants were excluded if they reported a family history of genetic conditions related to ASD (e.g., fragile X syndrome, tuberous sclerosis). All participants spoke English as their first language. Individuals were recruited from registries, local clinics, advocacy groups, and through advertisements that were widely dispersed throughout metropolitan and rural areas throughout the Midwest. All individuals provided informed consent, and procedures were approved by the University Institutional Review Board.

Demographic information is presented in Table 1. IQ was assessed using the Wechsler Abbreviated Scale of Intelligence (WASI), the Wechsler Intelligence Scale for Children (WISC)—Third or Fourth Edition, or the Wechsler Adult Intelligence Scale (WAIS)—Third or Fourth Editions (Wechsler, 1991, 1997, 1999, 2003, 2008), and participants were excluded if they had a VIQ < 80. As such, all participants included in this study were verbally fluent, with structurally complex spoken language. All participants were 10 years of age or older, since complex narrative production abilities are established by this age (Berman & Slobin, 1994; Kemper, 1984; Stein & Glenn, 1979; To et al., 2010; Westby, 1992). Participants with ASD had a previous clinical diagnosis of ASD, and the Autism Diagnostic Observation Schedule-Second Edition (ADOS-2; (Lord et al., 2012) and/or Autism Diagnostic Interview-Revised (ADI-R; Lord et al., 1994) was administered to confirm diagnostic status (American Psychiatric Association, 1994, 2013). All participants included in this study were administered ADOS modules 3 or 4 (i.e., fluent speech). To rule out ASD in the ASD Sibling Group, participants were screened for medical history of ASD and the ADOS-2 (Lord et al., 2012) was administered to participants under 18 years of age. Siblings who met criteria for ASD based on the ADOS-2 were excluded from the present study. Parents of individuals with ASD had at least one biological child with a clinical diagnosis of ASD. All efforts were made to recruit intact family units (e.g., parent-child dyads and ASD-ASD Sibling dyads), although in some cases the individual with ASD did not qualify for the present study due to exclusionary criteria, or parents were not available. Control participants were included if they had no personal or family history of ASD or related genetic disorders, as well as no family member within their nuclear family with a severe psychiatric disorder (e.g., bipolar disorder) or language-related delays.

**Table 1:** Demographic information

	ASD	ASD Siblings	Controls	ASD Parents	Parent Controls
	M(SD) Range	M(SD) Range	M(SD) Range	M(SD) Range	M(SD) Range
N (M/F)^a, b^	56 (47/9)	42 (15/27)	49 (25/24)	161 (64/97)	61 (24/37)
Age (years)^c^	18.34 (6.25)	17.06 (4.19)	19.04 (5.34)	46.35 (8.04)	42.49 (10.14)
	10.1–35.2	10.6–29.3	10.5–33.3	27.7–66.4	22.9–63.9
FSIQ^a, b^	106.75 (13.11)	115.71 (11.31)	117.71 (11.99)	111.44 (11.63)	114.82 (11.34)
	80–131	89–134	89–142	82–136	86–139
VIQ^a, b^	106.89 (14.10)	116.36 (13.47)	118.77 (11.72)	109.82 (11.49)	111.79 (12.64)
	84–146	82–146	92–142	80–132	82–138
PIQ^a, b^	104.18 (15.77)	112.5 (11.82)	113.52 (14.03)	110.02 (12.09)	113.51 (11.63)
	68–131	86–141	79–143	72–137	86–148
Word Count	414.05 (171.36)	410.95 (177.85)	407.78 (143.39)	482.24 (194.84)	468.30 (152.66)
	188–1029	171–1160	196–854	184–1165	210–909
Race	**N (%)**	**N (%)**	**N (%)**	**N (%)**	**N (%)**
White	38 (67.9%)	31 (73.8%)	35 (71.4%)	114 (70.8%)	57 (93.4%)
Black/African American	2 (3.6%)	1 (2.4%)	3 (6.1%)	9 (5.6%)	0 (0%)
Asian	1 (1.8%)	1 (2.4%)	9 (18.4%)	3 (1.9%)	1 (1.6%)
Native Hawaiian/Pacific Islander	0 (0%)	0 (0%)	0 (0%)	1 (0.6%)	0 (0%)
Biracial	2 (3.6%)	0 (0%)	1 (2.0%)	3 (1.9%)	0 (0%)
Other	1 (1.8%)	0 (0%)	1 (2.0%)	0 (0%)	0 (0%)
Not reported	12 (21.4%)	9 (21.4%)	0 (0%)	31 (19.3%)	3 (4.9%)
Ethnicity					
Hispanic	0 (0%)	0 (0%)	1 (2.0%)	6 (3.7%)	3 (4.9%)
Non-Hispanic	11 (19.6%)	16 (38.1%)	26 (53.1%)	137 (85.1%)	51 (83.6%)
Not reported	45 (80.4%)	26 (61.9%)	22 (44.9%)	18 (11.2%)	7 (11.5%)

^a^Significant difference between ASD and Control Groups

^b^Significant difference between ASD and ASD Sibling Groups

^c^Significant difference between ASD Parent and Parent Control Groups

### Narrative Procedures

#### Wordless Picture Book (PB)

*Frog Where Are You?* (“Frog Story”; Mayer, 1969) is a 24-page wordless picture book depicting the adventures of a boy and his dog while searching for a missing pet frog. The story is comprised of five main search episodes in addition to the introduction, plot establishment, and resolution. This picture book has been used extensively in research necessitating narrative elicitation (e.g., Bamberg & Marchman, 1991, 1990; Colle et al., 2008; Diehl et al., 2006; Lee et al., 2020; Losh & Capps, 2003; Losh & Gordon, 2014; Norbury & Bishop, 2003; Reilly et al., 2004; Sah & Torng, 2015; Tager-Flusberg, 1995; Thurber & Tager-Flusberg, 1993). In keeping with previous administration procedures (Lee et al., 2020), participants were asked to narrate the story page-by-page while viewing the stimulus on a Tobii T60 eye tracker. There was no time limit on the presentation of each page, and examiners advanced the pages as participants concluded speaking consistent with previous administration guidelines (e.g., Losh & Capps, 2003).

#### Transcription

All audio files of narration were transcribed following Systematic Analysis of Language Transcripts (SALT) (Miller & Chapman, 2008) conventions in either SALT or ELAN (Max Planck Institute for Psycholinguistics, 2022) software by trained transcribers who were trained to ≥ 80% word reliability for the narrative task and blind to group status. One third of files were randomly selected with equal representation of each diagnostic group to assess word-by-word agreement, and mean reliability across all files was 94%. Word count was used as a denominator to create proportion scores for certain variables (see below; i.e., Affect and Cognition, Causal Explanations) and was automatically calculated using Linguistic Inquiry Word Count (LIWC) software (Pennebaker et al., 2001). Prior to applying LIWC, transcripts were first cleaned to remove examiner utterances, followed by removal of participant utterance notations (e.g., “P” or “Participant:”), word repetitions, or transcription codes (e.g., aborted utterance symbols). Fillers were included in final word counts.

#### Hand-Coding

The narrative coding scheme was adapted from coding systems used in prior studies (Berman & Slobin, 1994; Losh & Capps, 2003; Reilly et al., 1990, 1998, 2004) and examined several key domains of narrative quality. The current hand-coding scheme was similarly designed to capture overall story structure and narrators’ understanding of the central search theme, as well as the ability to integrate protagonists’ perspectives into the story. See below for a detailed explanation of coding categories.

#### Story Structure

The structural framework of each narrative was captured by examining the presence of descriptions of key story elements across the narrative, including the setting, establishment of the plot, descriptions of the each of the story’s 5 main episodes where the boy encounters various obstacles as he searches for his missing pet frog, and the resolution. Each present key story element was tallied, and scores were summed with the 5 main episodes to create a total score for *Story Components-5 Episodes*, with a range of possible scores from 0 to 8.

Scores were also examined dichotomously to allow for a more global characterization of how narratives may be impoverished based on exclusion of key story elements from the story. For this variable, a 0 was assigned if any story component (i.e., setting, plot instantiation, search episodes, resolution) was missing, and a 1 was assigned if all elements were present. This variable was examined for the 5 main search episodes (*Missing Story Component- 5 Episodes).*

##### Setting

Presence of *Setting* included a description of the initial story setting, such as the physical or temporal setting (e.g., “There once was a boy and a dog and a frog and they were all together in a bedroom”).

##### Plot Establishment

Presence of the *Plot Establishment* included any mention of the story’s central search theme (i.e., the frog’s escape). For example, “Looks like frog is going to jump out of the jar”.

##### Search Episodes

Presence of descriptions of the story’s five main search episodes was assessed. See Table 2 for complete list of the search episodes.

**Table 2:** Five main search episodes and ten sub-episodes

Search Episode	Sub-Episode	Event
1		The boy and the dog search for the frog in the bedroom, and the dog falls out the window.
	1a	The boy and the dog search for the frog in the bedroom.
	1b	The dog falls out the window, and the boy rescues him.
2		The boy looks in a hole for the frog and finds a gopher, and the dog interacts with bees.
	2a	The boy looks in a hole for the frog and finds a gopher.
	2b	The dog discovers and interacts with bees.
3		The boy searches for the frog in a tree and is chased by an owl.
	3a	The boy searches for the frog in a tree and finds an owl.
	3b	An owl chases the boy.
4		The boy finds a deer, rides the deer with the dog, and they fall over a cliff.
	4a	The boy discovers a deer.
	4b	The boy rides a deer, and the boy and dog fall over a cliff.
5		The boy and dog land in a pond and look for the frog.
	5a	The boy and dog land in a pond.
	5b	The boy and dog search for the frog behind a log.

##### Resolution

Any description of the frog being found was coded as present. For example, “And then they found the frog, and the frog had found another frog friend”.

#### Thematic Coherence

Presence of the establishment and maintenance of the theme throughout the narrative.

##### Theme Establishment

This code captured descriptions that the frog was missing or that the boy and dog were searching for the frog. Any description of the initial theme was coded as presence of *Theme Establishment*. For example, “The frog went missing, and the boy went on a search for him”.

##### Theme Maintenance

Maintenance of the theme included descriptions throughout Search Episodes 1–5 where the boy and dog were searching for the frog (e.g., “Where are you, frog?”). Any description of the search theme throughout the narrative was coded as presence of *Theme Maintenance*.

#### Evaluation

The ability to enrich stories with the narrator’s perspective was assessed by noting instances of evaluative devices, including descriptions of *Affect and Cognition* and *Causal Explanations*.

##### Affect and Cognition

Descriptions of *Affective States and Behaviors* and *Cognitive States and Behaviors* (see below) were summed and examined as percent of total word count. Additionally, *Affective States and Behaviors* and *Cognitive States and Behaviors* were examined as a percent of all descriptions of *Affect and Cognition* to assess the proportion of usage of affect compared to cognition employed by participants. ***Affective States and Behaviors***. Descriptions of character’s emotions (e.g., happy, sad, scared) and behaviors related to their emotional states (e.g., smile, cower, kiss) were tallied. ***Cognitive States and Behaviors.*** This variable consisted of a tally of descriptions of character’s thoughts and internal states (e.g., think, realize) and behaviors related to those cognitive states (e.g., escape, sneak, hide).

##### Causal Explanations

Descriptions of *Causal Explanations of Behaviors* and *Causal Explanations of Affect and Cognition* (see below) were summed and examined as a percent of total word count. Causal explanations were coded for explicit language used to denote causality, such as “because”, as well as more subtle use of causal language including language markers such as “so”, “since”, “as a result”, “in order to”, and “therefore”. Additionally, *Causal Explanations of Behaviors* and *Causal Explanations of Affect and Cognition* were examined as a percent of all descriptions of *Causal Explanations* to assess the proportion of the types of causality used by participants. ***Causal Explanations of Behaviors.*** Descriptions of the cause or motivation of a behavior were tallied. For example, the following descriptions were coded: “The boy was running because he was being chased”; “The owl looked at the boy in order to see what was going on”. ***Causal Explanations of Affect and Cognition.*** Descriptions of the cause or motivation of an emotion or thought were tallied. For example, the follow descriptions were coded: “The boy was happy because he found the frog”; “Dog thought about the frog because he missed him”.

#### Secondary Hand-Coding Variables

Given the inclusion of clinically unaffected parents and siblings, secondary hand-coding variables were generated to capture more nuanced elements of narration that may be obscured in traditional hand-coding systems of narrative quality. Specifically, we detailed participants’ descriptions of the main search episodes, including the sub-episode level, as well as codes reflecting detail orientation (i.e., excessive use of detail and topic perseveration) throughout the story as follows:

##### Episodic descriptions

The quality of each episode description was assessed by breaking each episode into 2 sub-episodes (for a total of 10 sub-episodes). Key story elements (including 10 sub-search episodes) were tallied to create a total score for *Story Components-10 Sub-Episodes*, with scores ranging from 0 to 13. Scores were also examined dichotomously, such that if any story component (including sub-search episodes) was missing, participants received a score of 0 on *Missing Story Component-10 Sub-Episodes*, and a score of 1 was assigned if all components were present.

##### Detail and perseveration

To capture behaviors that may detract from narrative quality, we also evaluated narratives for the presence or absence of excessive detail and topic perseveration. Excessive detail was rated subjectively by coders as present or not present. Topic perseveration was coded as present when 3 or more mentions of one topic occurred throughout the narrative (e.g., including topics that are not the central theme, occur out of context, and do not contribute to the overall progression of the story).

#### Coding Reliability

Coders were trained on the coding scheme to at least 80% reliability across all codes and remained blind to participant diagnostic status. Approximately one third of transcripts were double-coded to assess reliability. Percent agreement was calculated for all nominal variables, while intraclass correlation coefficients (ICCs; Shrout & Fleiss, 1979) were calculated for all continuous variables. ICCs from 0.5 to 0.75 represent moderate agreement, 0.75-0.9 represents good agreement, and > 0.9 represents excellent agreement (Koo & Li, 2016). Agreement for nominal variables involving the presence of story structure and theme coherence met or exceeded 81% across groups, except in the proband groups for setting (78%) and sub-episode 2.1 (78%; boy discovers and interacts with gopher). Across groups, ICCs for all continuous variables were greater than or equal to 0.78.

### Gaze Procedures

Gaze was tracked from both eyes during the presentation of the PB. Areas of interest (AOIs) were established and applied to the PB stimulus (see below for details). Gaze data assessing percent duration of looking time to AOIs was previously published in ASD and ASD parents (Lee et al., 2020), albeit with a smaller sample than that included in the present study given ongoing data collection. Given new data distributions in the present study, a more stringent quality control procedure was implemented than the prior publication. Specifically, gaze data were analyzed if the ratio between the number of words to gaze track time was less than 4 words per second (compared to 5 words per second in Lee et al. (2020). See Lee et al. (2020) for a detailed explanation of quality control procedures.

AOIs in the PB task included all animate characters (i.e., boy, dog, frog, owl, deer, gopher, and bees), protagonists (i.e., boy, dog, and frog), protagonist’s focus of attention (i.e., where the protagonists were looking), and setting regions. AOIs were assessed for fixation count (a), fixation patterns (b), and fixation transitions (c) (see Nayar et al. (2022) and Table 2 for a detailed explanation of the following eye-tracking metrics):

- Each AOI was assessed for the number of fixations by calculating a proportion of the total fixations to each AOI as a percentage of all fixations.
- Perseverative and regressive fixation patterns were examined for each AOI. Perseverative fixations captured successive fixations within one AOI as a percent of all fixations, reflecting “sticky” visual attention. Regressive fixations included fixations back to a previously viewed AOI as a percent of all fixations, representing stimuli that was potentially the most prominent to the participant and may reflect inefficient processing of the gestalt of the picture book images, or a specific interest in AOIs previously examined.
- Fixation transitions were assessed between AOI types. AOIs were categorized into social (i.e., all characters) and non-social (i.e., setting) categories, and transitions were then characterized as either congruent (i.e., looking from one social AOI to another social AOI, or one non-social AOI to another non-social AOI) or incongruent (i.e., social AOI to non-social AOI, non-social AOI to social AOI). Fixation transitions were calculated as a percent of the total number of transitions. Fixation transitions provided information about the amount and types of visual exploration of the picture book.

#### Data Reduction for Gaze Variables

To examine the relationships between visual attention (as a construct) and narrative skills, all gaze variables were committed to data reduction methods based on prior work demonstrating their efficacy in reducing variables to one component or underlying construct (Nayar et al., 2022). Specifically, a principal component analysis (PCA) with a one-component solution was conducted for parent and proband/sibling groups separately for the below variables:

- Percent of fixations to social (i.e., animate AOIs) and nonsocial (i.e., setting) stimuli.
- Percent of perseverative fixations to social and nonsocial stimuli.
- Percent of regressive fixations to social and nonsocial stimuli.
- Percent of fixation transitions between social and nonsocial stimuli for all transition variables described above in (c).

The PCA-derived component explained 72% of the variance for all groups. Standardized loadings for each variable were ≥ 0.5 or ≤ − 0.5 (see *Supplementary Table 1* for loadings of each variable on the component). A regression component score was calculated for each participant, wherein higher ratings indicated greater attention to social stimuli.

## Analysis Plan

### Group Differences

For continuous narrative variables and all eye tracking metrics (outcome variables), linear mixed effects (LME) regression models were conducted with group (ASD, ASD Sibling, Control Groups) and relevant covariates (see below) included as fixed effects, and family included as a random effect to account for family nesting using the lmer (Bates et al., 2014) package for R (R version 3.6.1). Pairwise comparisons between groups were examined using the emmeans package for R and only interpreted when the overall model was significant. Analysis of variance (ANOVAs) were conducted for comparisons between parent groups. Binary narrative variables (i.e., presence/absence codes) were analyzed using 2 × 2 chi-square tests or a Fisher’s exact test in instances where > 20% expected cell values were < 5. Significance was set to *p* < .05; In addition to reporting p-values without corrections, we also report Benjamini-Hochberg corrected p-values (Benjamini & Hochberg, 1995) using a false discovery rate (FDR) of 0.10 to reduce false negatives and thereby potentially missing important effects.

### Assumptions Testing

Model assumptions were mostly met with the exception of one continuous variable, which appeared to have reduced normality per examination of quantile-quantile plots. Results for this variable (“Story Components- 5 Episodes”) using non-parametric methods were comparable to linear models; to allow for inclusion of covariates, results reported are those from the linear models only. There were no consistent outliers identified.

### Covariates

Age and/or IQ were considered as covariates if they met *both* of the following criteria: (1) group differences existed on the potential covariate, *and* (2) this potential covariate was theoretically or statistically associated with the outcome variables of interest. VIQ was included as a covariate in LME models involving ASD, Sibling, and Control Groups due to significant differences between the ASD Group and the Sibling and Control Groups, and known relationships between language abilities and narratives in children (Gabig, 2008). No covariates were applied to chi-square or Fisher’s exact test models due to limitations of the models, and no covariates were applied to analyses only involving eye tracking as VIQ did not consistently correlate with gaze outcome variables. No covariates were included in parent analyses.

### Correlation Analysis

Exploratory Pearson correlations were conducted within each group to assess relationships between the main narrative variables (i.e., *Story Components, Affect and Cognition, Causal Explanations*) and the eye-tracking component variable derived from the PCA. Significance was set to *p* < .05.

## Results

### Narrative Differences Across Groups. See Fig. 1

**Fig. 1:**
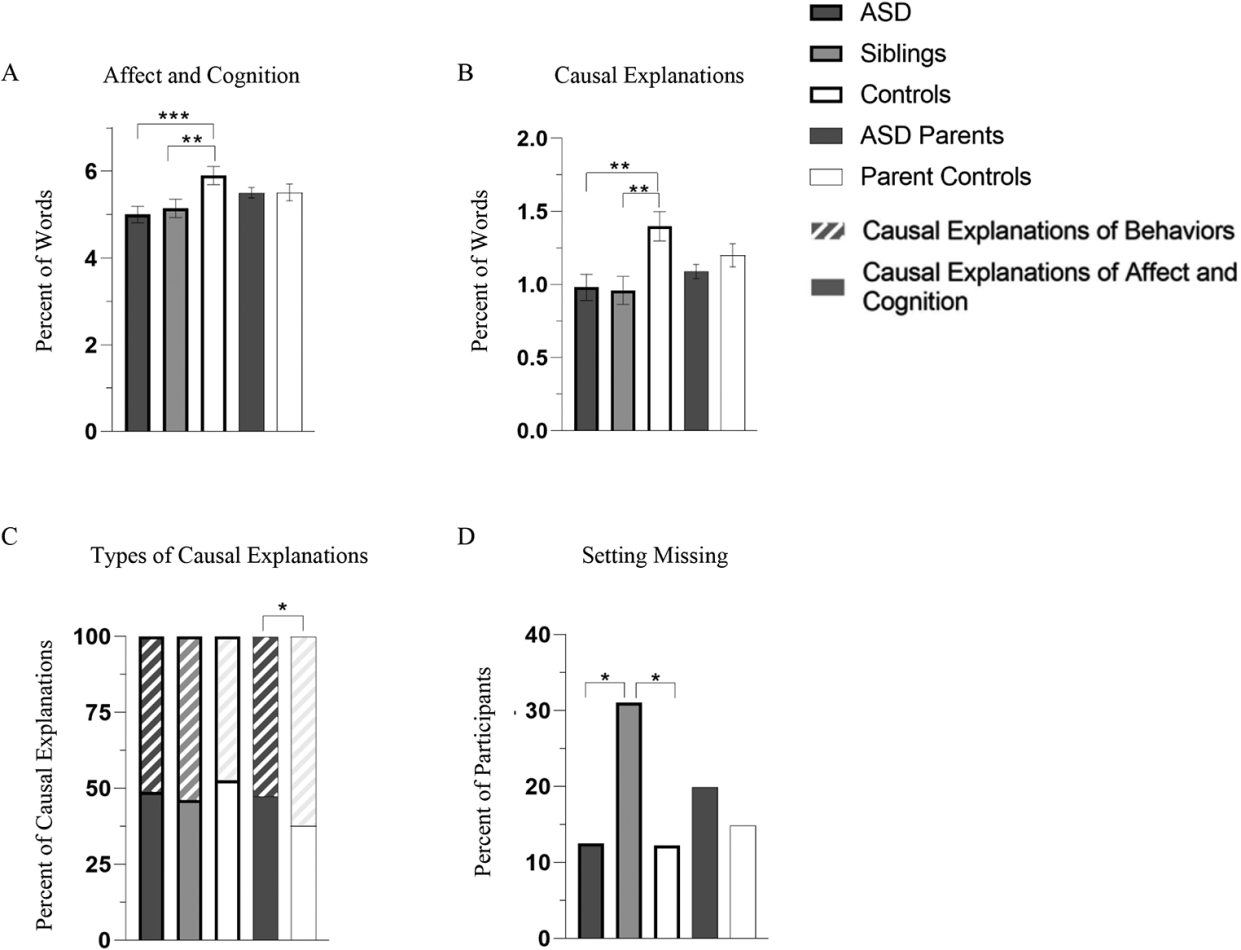
Narrative Differences. Narrative patterns depicting **A**) Frequency of descriptions of the character’s thoughts and feelings; **B**) Frequency of descriptions of causal explanations of affect, cognition, or behavior; **C**) Proportion of different types of causal explanations deployed; and **D**) The percent of participants excluding the setting story component from the narrative. *Note*. Errors bars are not included in Fig. 1C or 1D, which depict categorical variables

#### ASD and ASD Sibling Groups

Controlling for VIQ, the ASD and ASD Sibling Groups used fewer descriptions of affect and cognition (*t*(143) = 3.90, *p* < .001, β = − 0.38, adjusted *p* = .009; *t*(143) = 3.12, *p =* .002, β = − 0.38, adjusted *p* = .061, respectively) and fewer causal explanations (*t*(143) = 2.96, *p* = .004, β = − 0.29, adjusted *p* = .092; *t*(143) = 3.19, *p* = .002, β = − 0.30, adjusted *p* = .061, respectively) than the Control Group. Although no group differences emerged in thematic establishment or maintenance (*p*s ≥ 0.332, adjusted *p*s > 0.901), both the ASD and ASD Sibling Groups were more likely to omit story components compared to the Control Group (*χ*^2^(1) = 5.15, *p* = .023, adjusted *p* = .334; *χ*^2^(1) = 5.74, *p* = .023, adjusted *p* = .334, respectively). This difference appeared to be driven by a greater tendency for the ASD Sibling Group to omit the story setting compared to the ASD and Control Groups (*χ*^2^(1) = 5.03, *p* = .025, adjusted *p* = .334; *χ*^2^(1) = 4.79, *p* = .029, adjusted *p* = .334, respectively). No specific story component was significantly different from controls in the ASD Group (Fisher-exact *p*s ≥ 0.179, adjusted *p*s > 0.901). Secondary analyses also revealed no significant differences for either excessive detail or topic perseveration; Fisher-exact *p*s ≥ 0.258, adjusted *p*s > 0.901).

#### ASD Parent Group

Parent groups did not differ in rates of affective/cognitive statements (*F*(1, 220) = 0.001, *p* = .974, *ηp*^*2*^ = 0.000, adjusted *p* = 1.0) or general causal descriptions (*F*(1, 220) = 1.36, *p* = .245, *ηp*^*2*^ = 0.006, adjusted *p* = .584). However, groups differed in the *types* of causal explanations used, where ASD Parents used more causal explanations of character’s affect and cognition (*F*(1, 210) = 4.50, *p* = .035, *ηp*^*2*^ = 0.02, adjusted *p* = .434), and fewer causal descriptions of character’s behavior (*F*(1, 210) = 4.50, *p* = .035, *ηp*^*2*^ = 0.02, adjusted *p* = .434) compared to Control Parents. No group differences emerged in theme establishment/maintenance *(*Fisher-exact *p*s ≥ 0.361, adjusted *p*s > 0.654) or the total number of story components included (*F*(1, 220) = 3.48, *p* = .063, *ηp*^*2*^ = 0.02, adjusted *p* = .434). Secondary analyses showed no group differences in the inclusion of specific story components (Fisher-exact *p*s ≥ 0.065, adjusted *p*s > 0.434) or detail orientation (Fisher-exact *p*s ≥ 0.070, adjusted *p*s > 0.434).

### Gaze Differences Across Groups. See Fig. 2

**Fig. 2:**
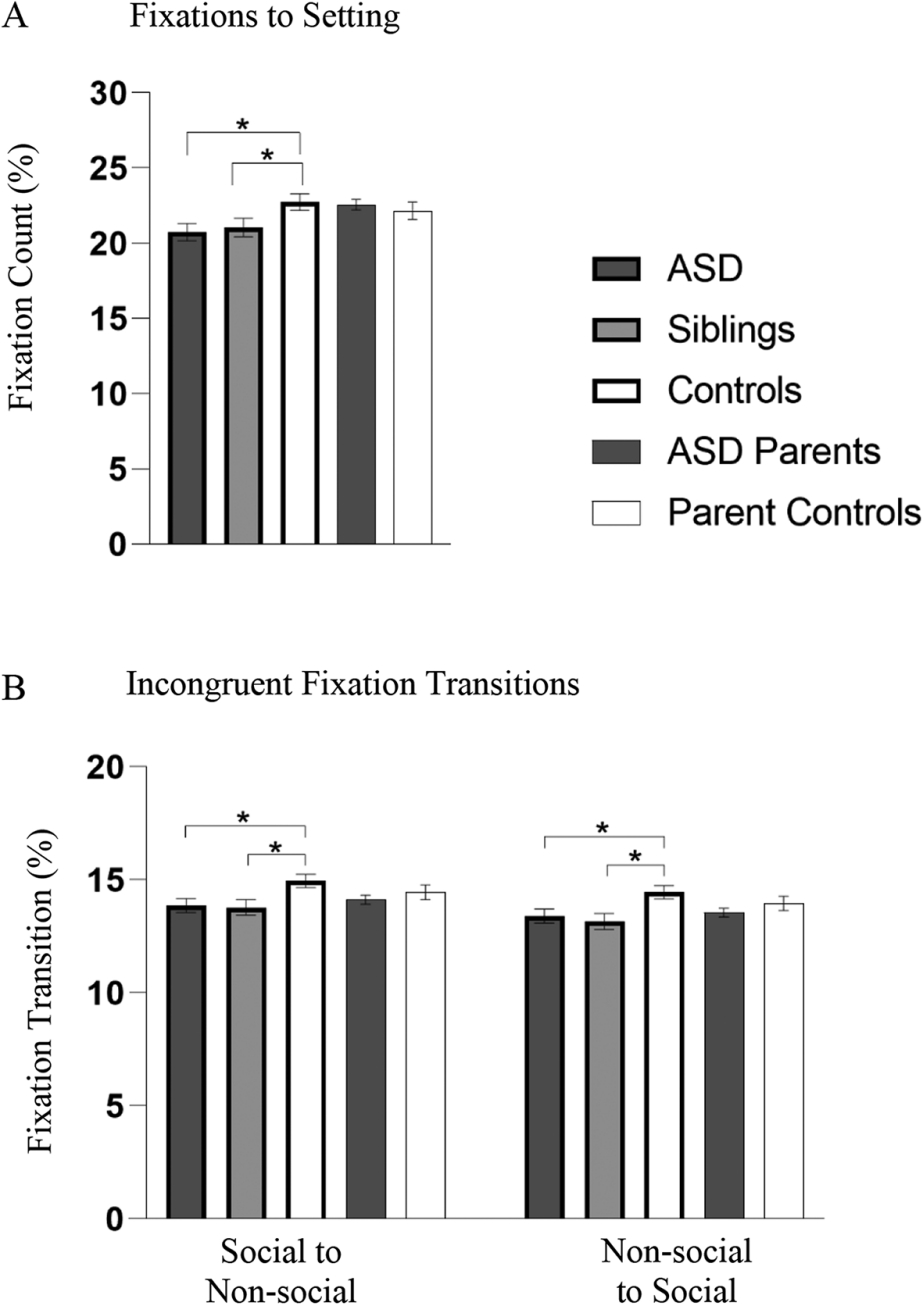
Gaze Differences. Gaze patterns across groups **A**) Number of fixations to the setting; **B**) Incongruent transitions between AOIs

#### ASD and ASD Sibling Groups

The ASD and ASD Sibling Groups made fewer fixations to setting AOIs (*t*(109) = 2.59, *p* = .011, β = − 0.27, adjusted *p* = .107; *t*(109) = 2.05, *p* = .043, β = − 0.21, adjusted *p* = .213, respectively). The Control Group made more incongruent transitions between AOIs than the ASD and ASD Sibling Groups (*t*(109) = 2.51, *p* = .014, adjusted *p* = .107, β = 0.27; *t*(109) = 2.72, *p* = .008, β = 0.28, adjusted *p* = .107, respectively), including more transitions from social to nonsocial AOIs (*t*(109) = 2.56, *p* = .011, β = − 0.27, adjusted *p* = .107; *t*(108.5) = 2.47, *p* = .015, β = − 0.26, adjusted *p* = .107, respectively) and nonsocial to social AOIs (*t*(109) = 2.31, *p* = .023, β = − 0.24, adjusted *p* = .146; *t*(109) = 2.67, *p* = .009, β = − 0.28, adjusted *p* = .107, respectively).

#### ASD Parent Group

No gaze differences emerged in the parent groups (*p*s > 0.187, adjusted *p*s > 0.871).

### Relationships Between Narrative and Gaze

All gaze associations were conducted with the PCA-derived gaze metric of social attention.

#### ASD and ASD Sibling Groups

There were no associations between narrative ability and gaze in the ASD (*r*s < |0.30|, *p*s ≥ 0.070, adjusted *p*s > 0.314) or ASD Sibling Groups (*r*s < |0.32|, *p*s ≥ 0.079, adjusted *p*s > 0.314). In the Control Group, greater social attention was associated with fewer causal explanations during narration (*r* = − .34, *p* = .027, adjusted *p* = .314). There were no significant correlations between gaze and the number of story components or descriptions of affective and cognitive states and behaviors (*r*s < |0.23|, *p*s ≥ 0.146, adjusted *p*s > 0.313).

#### Parent Groups

In the ASD Parent Group, increased social attention was associated with more descriptions of *Affect and Cognition* (*r* = .21, *p* = .030, adjusted *p* = .240). No correlations between social attention and *Story Components and Causal Explanations* were found (*r*s < |0.13|, *p*s ≥ 0.209, adjusted *p*s > 0.674), nor were gaze and narration variables significantly correlated in the Parent Control Group (*r*s < |0.19|, *p*s ≥ 0.263, adjusted *p*s > 0.674).

## Discussion

This study aimed to deeply characterize how narrative skills are influenced in ASD and first-degree relatives of autistic individuals to understand the social-communicative profile impacted by ASD-related genetic variation. We also aimed to delineate narrative and potentially related attentional skills that may inform targeted social-communication interventions for autistic individuals. Prior work had shown robust differences in narrative use in ASD, and more subtle differences among parents of autistic individuals. In this study, we aimed to extend this work by examining narrative in siblings of autistic individuals as well, by applying a detailed hand coding system to assess fine-grained narrative features and potential associations with visual attention during storytelling. Findings revealed some important parallel patterns in the ASD and sibling groups and more subtle differences in the ASD parent group, with different patterns of causal explanations emerging among all groups, discussed further, below.

The types of language used to reflect internal states of characters (i.e., cognitive and affective states) and to establish causal connections between elements of the story were examined as an important index of narrative coherence, complementing the more global storytelling skills such as inclusion of story episodes and establishing a story’s theme. For both the ASD and ASD sibling groups, findings revealed a decreased use of cognitive and affective state language and causal language (which also withstood corrections for multiple comparisons). Similar gaze patterns in ASD and ASD sibling groups were also found, which could inform the mechanisms underlying observed narrative differences. Both groups were also more likely to omit key story components (i.e., setting, instantiation, sub-episodes, or resolution) relative to controls, suggesting a reduced tendency to attend to salient aspects of the storybook. Findings showing diminished visual exploration of the storybook scenes in the ASD and ASD sibling groups (as reflected by less dynamic gaze transitions that were focused within congruent stimuli—i.e., social to social stimuli, nonsocial to nonsocial stimuli) offer some insight into neurocognitive mechanistic processes that may underly narrative differences. In this vein, it is possible that aspects of the story that were not mentioned in narratives may have resulted from inattention to those events visually, and thus less salient or efficient encoding. Marked differences detected in the ASD Sibling Group specifically revealed a tendency to omit description of the story’s setting (while the ASD group omitted details across any key story component suggesting more general differences in story narration than those observed among siblings). Converging eye-tracking results showed reduced attention to the setting during narration in the ASD Sibling Group. For ASD parents, findings were more nuanced, revealing a subtle difference in how parents deployed causal language throughout their narratives. Specifically, whereas ASD parents used causal language with similar frequency as controls, they tended to focus more on explaining characters’ thoughts and emotions than interpreting their behaviors, relative to controls. Differences in the use of causal language have been noted repeatedly in prior studies of ASD (Diehl et al., 2006; Losh & Capps, 2003, 2006; Tager-Flusberg & Sullivan, 1995), highlighting this finding as potentially significant and revealing of core differences in narrative associated with ASD genetic likelihood.

The patterns of differences observed in these key aspects of narrative (most obviously in the ASD Group, followed by the ASD Sibling Group) are intriguing and could suggest that these narrative skills reflect heritable traits influenced by ASD-related genetic variability. Such traits could be useful to study to help parse out gene-behavior associations in ASD, particularly given the robust literature on the biological basis of visual attention (Constantino et al., 2017; Eckstein et al., 2017). Observed narrative and gaze differences could also be used to help reduce heterogeneity in biological studies, by stratifying families into phenotypically more homogeneous subgroups where meaningful signals may be more easily detected. Of course, narrative skills are also known to be influenced by social and communicative environments, including parental discourse styles (e.g., Haden et al., 1997). Bi-directional relationships are also possible, where autistic individuals’ narrative styles may be influential in siblings’ developing narrative skills (although, no notable difference in average age was observed in the current sample). However, evidence that language-related differences are observed in ASD parents during childhood (based on retrospective academic testing data; Losh et al., 2017), well before they would become a parent of an autistic child, in addition to the extensive literature documenting pragmatic differences in the broad autism phenotype (BAP; thought to reflect underlying genetic liability to ASD), ASD-related genetic influences on siblings’ and parents’ narratives skills are important to consider.

Narrative differences observed in the ASD and ASD sibling groups may also have clinical implications. Whereas variability in narrative style is an important contributor to the complex distinctions that make individuals and cultures unique, it is important to consider the differences observed in the ASD and ASD sibling group in the context of the importance of narrative as primary social communication tool. Narrative is among the most common forms of social communication, used to share experiences with others in meaningful ways and build relationship based in part on lived experiences that are co-constructed and shared through narrative (Bruner, 1990; Ochs & Capps, 2009). The specific narrative differences observed in the ASD Group in particular, with differences also observed in the ASD Sibling Group, all serve important functions, and their absence could contribute to challenges in social communication. For instance, inferring characters’ thoughts and feelings and discussing how events are causally related in narrative are vital to conveying the importance of narrated events and for connecting meaning to characters’ experiences (Diehl et al., 2006). Similarly, introducing a narrative topic through setting description is important for anchoring the central plot, setting the stage for the unfolding story, and engaging listeners with background for what is to come. Omission of this important component can undermine the effectiveness of a narrative, resulting in more factual description than meaningfully connected narrative. Findings are therefore important to consider in the context of broader social communication differences that characterize ASD, and the more subtle and specific social communication challenges that may be present for siblings, even in the absence of a clinical diagnosis of ASD.

Findings of limited differences in the parent groups are perhaps not surprising, given the subclinical presentation of the BAP-related pragmatic differences documented previously among parents, and that the highly structured nature of this narrative task (e.g., unfolding with serially ordered events and page-by-page presentation offering scaffolding for participants to develop their stories) may have obscured more subtle differences in narrative skill. Prior differences reported in parent groups have indeed been quite subtle, and it may be that the picture book task in this study was not sufficiently challenging to elicit more obvious differences that had been reported in prior work using more complicated narrative tasks (e.g., Landa et al., 1991; Lee et al., 2020). Different patterns in parents’ use of types of causal language stand in contrast to the otherwise intact narrative skill observed across other narrative variables. When considered together with similar differences detected in ASD and in siblings, and with prior reports of differences in causal language during narration and conversation in ASD (Diehl et al., 2006; Losh & Capps, 2003, 2006; Tager-Flusberg & Sullivan, 1995), causal attributions may be considered a critical narrative skill impacted by ASD-related genetic variation. Although the ASD parent group produced overall causal language at rates similar to controls, they tended to focus more on causal explanations of affect and cognition, and less on causal explanations of behavior, relative to controls.

It may be that parents of autistic individuals find the integration of the character within the larger story to be more salient, thereby leading them to discuss them more in their narratives. This possibility is supported by gaze patterns which showed associations between greater descriptions of affect/cognition and greater social attention. In another narrative task including a portion of the participants reported here, and examining more coarse-grained gaze variables, Nayar et al. (2022) identified differential patterns of fixations, where the ASD group showed elevated perseverative attention towards non-social information while parents showed reduced perseveration towards social information. As with causal language differences, such patterns could represent a pathoplasticity, where biologically meaningful ASD-related traits manifest variably across the spectrum of the condition from clinical to sub-clinical levels, and may permeate across cognitive functions from gaze to language.

### Limitations and Future Directions

The present study assessed narrative abilities in individuals with ASD and their siblings and parents within a highly structured task, which may have limited power to detect more subtle differences present in relatives. Future studies should further assess narrative skills in less structured tasks with different social and cognitive demands (e.g., semi-structured conversations) (Goodkind et al., 2018; Klusek et al., 2014b) to fully understand genetically meaningful phenotypes associated with narrative deficits in ASD. Further, whereas the current study expanded on previous literature and developed a detailed hand-coding system with more in-depth coding of features such as detail orientation and classification of sub-episodes to deeply characterize participants’ narratives (Losh & Capps, 2003), results did not reveal difference in those more detailed codes, with primary differences instead found with variables coded with the less detailed coding systems used in prior work. This finding is important in guiding future work, suggesting that the highly time intensive coding conducted here may not be necessary to capture clinically and potentially biologically meaningful differences in narrative in ASD and first-degree relatives. Analyses involving categorical variables (and specifically findings pertaining omission of the setting) did not include relevant covariates and future analyses may consider an alternative coding system to ensure inclusion of covariates.

Relatedly, although patterns of differences in narrative and gaze were related conceptually (and were often of marginal significance and thus not reported), few significant narrative-gaze associations emerged across groups. It is possible that the eye-tracking metrics applied across the entirety of the picture book task masked potentially meaningful and important patterns of gaze that could unfold over the course of time (e.g., see Nayar et al., 2018, 2022). Future efforts may benefit from application of a temporal longitudinal analyses to gaze patterns that are synchronized to speech to robustly capture relationships more reflective of the dynamic nature of visual attention. Additionally, given findings of different pragmatic and narrative skills among males versus females with ASD (Boorse et al., 2019; Bylemans et al., 2023; Conlon et al., 2019; Kauschke et al., 2016), as well as sex-linked differences in pragmatic functions in the BAP (Nayar et al., 2021), it will be important for future studies to include well-powered groups of females to detect possible sex differences in narration and gaze in ASD and among relatives. Although several racial identities were included in this study, most participants self-identified their race as “White”. As such, future work assessing narrative ability in autism should involve a more diverse sample of participants, encompassing different racial/ethnic backgrounds as well as other dimensions of diversity, such as sex/gender, culture, and language. Finally, due to the research questions addressed, the current study included a sample of participants who were verbally fluent with a VIQ of at least 80, and studying participants with more limited verbal abilities is an important next step to understand the generalizability of findings to individuals with a wider range of cognitive and verbal abilities.

In sum, by characterizing narrative skills in ASD and among first-degree relatives using an extensive hand-coding system and collecting gaze during narration, this study captured important features of narrative skills impacted across the spectrum of ASD genetic influence. Important parallel patterns were detected in ASD and in siblings, with more subtle patterns of narrative differences observed in parents, which together, may help to refine the profile of social communication skills impacted by ASD-related genetic variation.

## Data Availability

The datasets used and/or analyzed during the current study are available from the corresponding author on reasonable request.
